# Supplementary material for: Influenza vaccine hesitancy among healthcare workers in a Northeastern province in Thailand: Findings of a cross-sectional survey
Source: PLoS One. 2024 Sep 19;19(9):e0310761. doi: 10.1371/journal.pone.0310761 (PMC11412645; doi:10.1371/journal.pone.0310761)
Supplement: S1 Table — (PDF) [file pone.0310761.s001.pdf]

Supplemental Table 1. HCW Influenza Vaccine Hesitancy Determinant Items (5 point Likert scale):

Determinants	Items
**a) Contextual factors:**	
Influence of media/social media	• The reports in the media/ on social media have made me reconsider the choice to receive influenza vaccine.<br>• I trust the negative rumors about vaccines in the social media or other sites in the internet.
Politics/policies	• The reports in the media/ on social media have made me reconsider the choice to receive influenza vaccine.<br>• I trust the negative rumors about vaccines in the social media or other sites in the internet.
Pharmaceutical industry influences	• I trust the motives of pharmaceutical industry.<br>• I trust pharmaceutical companies to provide safe and effective influenza vaccines.
Trust in Health system	• I trust the information I receive about influenza vaccine.<br>• The information on side-effects following influenza vaccination is discussed openly by the authorities.
**b) Individual/group influences:**	
Experience with past vaccination	• I have, at times, not accepted an influenza vaccination for myself when it was offered.<br>• I or someone I know had a bad reaction to influenza vaccine in the past which made me reconsider getting influenza vaccine.<br>• My experiences with pain with past immunization prevent me from being vaccinated against influenza.
Beliefs and attitudes about influenza and vaccination	• Influenza infection is needed to build natural immunity and that vaccines destroy the important natural immunity against influenza.<br>• Influenza vaccine can overload the immune system.<br>• There are better ways to prevent influenza infection than by an influenza vaccine.
Vaccination as a social norm	• It is important for every HCW to get the recommended influenza vaccines for themselves every year.<br>• It's important for HCW to be vaccinated against influenza to protect those high-risk patients that cannot get vaccinated<br>• I feel social pressure to receive an annual influenza vaccine.

Influence of other healthcare professionals	• My healthcare provider/vaccinator had ever advised that influenza vaccine was not necessary or had too many side effects.<br>• My healthcare provider/vaccinator has hesitated to recommend or administer influenza vaccines to high-risk people due to their own doubts regarding the influenza vaccine.
**c) Influenza vaccination-specific issues**	
Perceived Risk/benefit	• Influenza is not a serious or severe enough disease that requires annual vaccination for prevention.<br>• I am concerned that I might have a serious side effect from an influenza vaccine.<br>• I have decided against influenza vaccine because I think I am not at a high risk of influenza infection.<br>• Influenza vaccine can prevent severe episodes of influenza.
Risk of adverse events due to vaccination	• Influenza vaccine is safe for myself and for other high risk groups.<br>• There is adequate safety information regarding annual influenza vaccines.<br>• The system in Thailand can track adverse reactions or side effects to influenza vaccinations
Fear of painful injections	• The fear of pain or fear of needles make me hesitant to receive influenza vaccine.<br>• I trust my vaccinator can safely administer influenza vaccine to me.
Access to vaccines in the hospital	• Access to influenza vaccination easy/convenient in my hospital<br>• There are adequate number of influenza vaccines in my hospital during vaccine campaign to cover all high-risk population in the area.<br>• There are barriers for receiving influenza vaccine in time at my hospital.<br>• The vaccines that I need to pay for myself are more effective than the free-of-charge vaccines provided at my hospital by the government.
