# Supplementary material for: Influenza vaccine hesitancy among healthcare workers in a Northeastern province in Thailand: Findings of a cross-sectional survey
Source: PLoS One. 2024 Sep 19;19(9):e0310761. doi: 10.1371/journal.pone.0310761 (PMC11412645; doi:10.1371/journal.pone.0310761)
Supplement: S2 Table — (PDF) [file pone.0310761.s002.pdf]

Supplemental Table 2. Response rates from each of the study hospitals

Location	Expected respondents (N)	Respondents (n)	Response rate (%)
**Nakhon Phanom Hospital**	100	100	100.0
**That Phanom Hospital**	50	50	100.0
**Phonsawan Hospital**	50	50	100.0
**Plapak Hospital**	50	49	98.0
**Na Kae Hospital**	50	49	98.0
**Wang Yang Hospital**	50	40	80.0
**Overall**	**350**	**338**	**96.6**
