# Supplementary material for: Influenza vaccine hesitancy among healthcare workers in a Northeastern province in Thailand: Findings of a cross-sectional survey
Source: PLoS One. 2024 Sep 19;19(9):e0310761. doi: 10.1371/journal.pone.0310761 (PMC11412645; doi:10.1371/journal.pone.0310761)
Supplement: Attachment — Submitted filename: Response to Reviewers.docx [file pone.0310761.s005.docx]

**Response to Reviewers’ comments**

Dear Dr. Fadi Aljamaan,

We would like to thank you and the reviewers for reviewing our submission and providing us with valuable suggestions for improving the manuscript.

We have incorporated the reviewer’s feedback into our revised manuscript version. We have outlined our response to the journal comments and the specific comments of the reviewers, along with the changes made in the following section:

**Journal Requirements:**

1) Please ensure that your manuscript meets PLOS ONE's style requirements, including those for file naming. The PLOS ONE style templates can be found at

<https://journals.plos.org/plosone/s/file?id=wjVg/PLOSOne_formatting_sample_main_body.pdf> and

<https://journals.plos.org/plosone/s/file?id=ba62/PLOSOne_formatting_sample_title_authors_affiliations.pdf>

Author response:

We have made some formatting changes to the title page and the body of the paper to meet PLOS ONE's style requirements. We have also edited the filenames as instructed in the templates.

2) Please include captions for your Supporting Information files at the end of your manuscript, and update any in-text citations to match accordingly. Please see our Supporting Information guidelines for more information: http://journals.plos.org/plosone/s/supporting-information.

Author response:

We have edited the in-text citations of supporting information files and added their captions after references in the manuscript, according to PLOS ONE's style requirements.

**Reviewer comments:**

**Reviewer #1:** Thank you for submitting this work titled Influenza vaccine hesitancy among healthcare workers in a Northeastern province in Thailand: Findings of a cross-sectional survey

Author response:

We thank Reviewer #1. However, we did not receive the full comments despite waiting till the last day to submit the revised version. We believe that Reviewer #1 generally liked our work and had no issues with the paper. We extend our gratitude to Reviewer #1.

**Reviewer #2:** Thank you for this important work. Please, find my comments below.

Author response:

We would also like to thank Reviewer #2. We have tried to address each of his comments below:

1. On what basis did the authors include those who “accepted the vaccines but were unsure” as HESITANTS. Can you cite any reference/guideline/consensus statement for this? And what is the actual number of the study participants who fall into this category? Lumping those who indicated acceptance but were unsure cannot be accurate since the concept of vaccine acceptance/hesitancy itself is dynamic being determined by many other external influences. And I think this may be the reason why only 3 factors were significantly associated with hesitancy in the final adjusted model, with the age factor being an odd finding with respect to the overwhelming findings of previous studies.

Author response:

We thank the reviewer for highlighting this crucial distinction of this study. We included those who “accepted the vaccines but were unsure” as “hesitants” in our study beacuse this group has doubts or concerns about the vaccine but still get the vaccine reluctantly. This is a clear demarcation from the dichotomy of vaccine acceptance or non-acceptance as vaccine hesitancy.

This distinction is supported by literature (e.g., Dube et al., 2013; Bussink-Voorend et al., 2022; MacDonald, 2015). For example, Dube et al. (2013) mention that vaccine-hesitant individuals may accept all recommended vaccines in a timely manner, but still have significant doubts in doing so. Similarly, Bussink-Voorend et al. (2022) report that people may accept vaccines with or without hesitation or reject vaccines with or without hesitation. Importantly, the SAGE working group mentions, "Hesitancy is set on a continuum between those that accept all vaccines with no doubts, to complete refusal with no doubts, with vaccine-hesitant individuals the heterogeneous group between these two extremes. Hesitant individuals encompass a wide range of people who differ from the very small percentage who refuse all vaccinations and have no doubts about doing this (MacDonald, 2015).

Additionally, Larson et al. (2013) opine that vaccine hesitancy is, by nature, a state of indecision and reluctance. In this conceptualization, vaccine hesitancy is mainly described as ‘doubts,’ ‘concerns’, and ‘reluctance’ regarding vaccination. Many authors have argued that vaccine hesitancy is a psychological state rather than a behavior of getting the vaccine (Bedford et al., 2018; Betsch et al., 2018; and Deml et al. 2020). We have added this information to the introduction to make our stance clearer (lines 100-106 in the track changed file) *(all line numbers relate to the track changed file in All Markup mode)*

The actual number of the study participants who fall into this category has been mentioned in the results section under the subheading of “Influenza vaccine hesitancy among the participants” as “On closer inspection, most of the hesitant HCWs were “hesitant compliers” who had doubts about the influenza vaccines but still accepted them (172; 87.3%)” (line 335-336), and also shown clearly in Fig 2.

While we agree that this group might have been influential in significantly affecting the 3 factors in the final adjusted model, we believe the results are indicative of the scenario at Nakhon Phanom province, especially after corroborating with the qualitative findings (to be published in a future paper).

References:

Bedford, H. et al. Vaccine hesitancy, refusal and access barriers: the need for clarity in terminology. Vaccine 36, 6556–6558 (2018).

Betsch, C. et al. Beyond confidence: development of a measure assessing the 5C psychological antecedents of vaccination. PLoS ONE 13, e0208601 (2018).

Bussink-Voorend, D. et al. A systematic literature review to clarify the concept of vaccine hesitancy. Nat Hum Behav 6, 1634–1648 (2022).

Deml, M. J. et al. ‘Problem patients and physicians’ failures’: what it means for doctors to counsel vaccine hesitant patients in Switzerland. Soc. Sci. Med. 255, 112946 (2020).

Dubé, E. et al, Vaccine hesitancy, Human Vaccines & Immunotherapeutics, 9:8, 1763-1773 (2013).

Larson, H. J. Negotiating vaccine acceptance in an era of reluctance. *Hum. Vaccin. Immunother.* **9**, 1779–1781 (2013).

MacDonald, N.E. Vaccine hesitancy: Definition, scope and determinants. Vaccine. 33:4161-4 (2015)

2. Can the authors specifically define what they mean by “acceptance”, whether, for example, the term includes those already vaccinated plus unvaccinated who are willing to be vaccinated, or whether the term refers only to unvaccinated who are willing to be vaccinated?

Author response:

We included all HCW willing to be vaccinated with the influenza vaccine without any doubt as “acceptance”, irrespective of their last year or current year vaccination status (Fig 1).

3. Can the authors provide a reference for their affirmative statement “…acceptance of vaccination by HCWs themselves is, therefore, also an important determinant of influenza vaccine uptake in the populations that they serve”

We have added two references that report that vaccinated HCWs are more likely to recommend vaccination to others (Paterson et al., 2016), and HCWs’ recommendations are an important cue to action for patients such as pregnant women to receive an influenza vaccine (Ditsungnoen et al., 2016) (line 91).

4. Please cite the webpage/URL for your statement in line 101-103, “However, in 2018, regional reports showed that HCW influenza vaccine coverage had plummeted to below 30% in some regions (Department of Medical Services [DMS], MOPH)”.

The regional reports have been removed from the DMS website. We have included the report's Excel data file as supporting information (S1 File) (line 126 and 141).

5. If the “mixed-methods study”, which the present study is said to be part of by the authors has been published, please cite it were you said this.

The mixed-methods manuscript is under preparation and has not been submitted to journal or published yet.

6. Why would the authors say “As there was no prior estimate of influenza vaccine hesitancy among Thai HCWs” in line 160 under Methods section, while in line of the introduction, they wrote “A study 105 revealed that only 60% of Thai HCWs were willing to receive the pandemic influenza (H1N1) vaccine [21]”? Isn’t this a self-contradiction?

The concept of vaccine hesitancy was not in use before this study in Thailand, as it became readily available only after the WHO definition in 2015. The study cited in the introduction is from 2011, where the HCWs’ acceptance/non-acceptance of pandemic influenza vaccine was measured as a dichotomous variable, which is not entirely the same as “vaccine hesitancy”. This has also been elaborated on above in the response to the first question.

7. In your methods, please describe the tool used to assess knowledge, specify whether it is adapted or adopted, and whether it has been validated.

The tool used was a subset of questionnaire from a previous study among Thai physicians (Praphasiri et al., 2017). This reference has been added in the methods section (line 251-252).

8. In the analysis, have the authors evaluated model fitness? If so, please state which were used, and if not, please explain why.

While we conducted the Hosmer–Lemeshow test to determine the goodness of fit of the final logistic regression model, we did not report it as Hosmer and Lemeshow (2013) themselves do not recommend using their test unless the sample size is at least 400, because of insufficient power. Our sample size was 331.

Nonetheless, we report it here:

*Hosmer–Lemeshow chi-squared (8) = 14.929, p-value = 0.061*

The p-value is > 0.05, indicating that there is no significant lack of fit for the logistic regression model. The model fits the data adequately, although the p-value is close to the threshold of 0.05, suggesting a marginal fit. Alternatively, the Nagelkerke R Square value was 0.245, indicating a moderate relationship between the predictors and the outcome.

References:

Hosmer Jr, D. W., Lemeshow, S., & Sturdivant, R. X. (2013). Applied logistic regression. New York: Wiley.

9. Given that this study was conducted during the early phase of the COVID-19 pandemic, a period that has saw an unprecedented levels of rise in vaccine hesitancy and antagonism, have the authors considered a spillover of this COVID-19 vaccine-related hesitancy into influenza vaccine and all other vaccines?

We agree with the reviewer that the early phase of the COVID-19 pandemic saw unprecedented levels of rise in vaccine hesitancy and antagonism globally. We could not detect the how the COVID-19 in Thailand might have affected some HCWs’ influenza vaccine decision-making as the questionnaire was designed in the pre-COVID period. Nonetheless, our qualitative in-depth interviews showed that few Thai HCW were afraid of the looming COVID pandemic which had not hit the shores of Thailand till then, so much so that were willing to get the influenza vaccine, perceiving some extended benefits to the early flu like symptoms of COVID. However, as this was not captured in the quantitative survey, we haven’t reported in this paper. We have kept it for reporting in the subsequent mixed methods paper that will be submitted later this year.

10. In your Discussion section, please clarify why you would say that there are “no prior estimates of the prevalence of influenza vaccine hesitancy among the HCWs” despite citing acceptance rates of the same vaccine among the same study population in the same country. Note subtracting these reported acceptance rates gives you the hesitancy rates, right?

Similar to the response above to question 6, we opine that vaccine hesitancy is not equal to vaccine non-acceptance. Simply subtracting the acceptance rates does not do justice to the notion of vaccine hesitancy. Vaccine hesitancy is more dynamic and encompasses a wide range of people who differ from the people who accept or refuse the vaccine without having any doubts about doing so. We have edited the discussion slightly to make it clearer in lines 421-427:

*“Although these comparisons are limited due to differences in vaccine hesitancy conception, study sites, and the HCW definition, which was expanded to include all types of HCW working in the hospital in this study as opposed to only doctors or medical service providers in the past studies, they indicate that HCW in Nakhon Phanom may be more hesitant towards influenza vaccines than normally expected.”*

11. The probable reason(s) for higher odds of hesitancy in HCWs age >50years (who at higher risk of the infection than those below) need to be better hypothesized in your Discussion. I think this finding may be related to the inaccurate analysis that lumps “those who accept but were unsure” as hesitants.

We thank the reviewer for focusing on older age being associated with increased hesitancy. While studies in other parts of the world show that younger folks are more hesitant to vaccines, the same was not true among Thai HCWs in this rural province. We do not think it is due to an inaccurate analysis. On the contrary, our qualitative findings explain why this was the case in this province. Our qualitative findings suggest that seniority in rural Thailand's highly-regarded public health service might have given the older HCWs more power to resist the vaccination policy and approaches of vaccinators. We have explored this in the discussion section as the social capital of older HCWs, who by the virtue of their experience and higher position in the hospital authority, could openly display their vaccine hesitancy, which may have been derived due to difficulties in past vaccinations, while the younger HCWs had to comply to the vaccination policy at the hospital.

We hope that our changes will have addressed all the comments and meet with the approval of the reviewers.

Thank you for your kind consideration, and we look forward to hearing from you soon.

Best regards,

**Prabda Praphasiri,**

Corresponding Author
