# Supplementary material for: Influenza vaccine hesitancy among healthcare workers in a Northeastern province in Thailand: Findings of a cross-sectional survey
Source: PLoS One. 2024 Sep 19;19(9):e0310761. doi: 10.1371/journal.pone.0310761 (PMC11412645; doi:10.1371/journal.pone.0310761)
Supplement: Attachment — Submitted filename: Response to Reviewers.docx [file pone.0310761.s006.docx]

**Response to Reviewers’ comments**

Dear Dr. Sirwan Khalid Ahmed,

We would like to thank you and the reviewers for reviewing our revised submission and providing us with valuable suggestions for improving the manuscript.

We have added my official email address to the title page and incorporated the reviewer’s feedback into this version of our manuscript. We have outlined our response to the journal requirements and the specific comments of the reviewers, along with the changes made in the following section:

**Journal Requirements:**

Please review your reference list to ensure that it is complete and correct. If you have cited papers that have been retracted, please include the rationale for doing so in the manuscript text, or remove these references and replace them with relevant current references. Any changes to the reference list should be mentioned in the rebuttal letter that accompanies your revised manuscript. If you need to cite a retracted article, indicate the article’s retracted status in the References list and also include a citation and full reference for the retraction notice.

Author response:

We have reviewed our reference list and we vouch that it is complete and correct. We have also checked the retraction status of each paper cited. None of the cited papers have been retracted.

**Reviewer comments:**

**Reviewer #2:** Thank you for revising this work. I have gone through the revision and its supporting responses and I agree with them. I have no new comments. Looking forward to seeing the published work online.

Author response:

We wholeheartedly thank Reviewer #2. His comments made us reflect on the nature of vaccine hesitancy and improved our paper significantly.

**Reviewer #3:**

2. How the author makes sure that the participants were not biased towards specific answer

3. How the author makes sure that the participants were not disengaged while answering.

4. How the author makes sure about reliability of result

5. Author need to mention reliability and validity of tools used in the study

6. Author need to mention response rate

Author response:

We would also like to thank Reviewer #3. We have tried to address each of the questions/comments below:

2. How the author makes sure that the participants were not biased towards specific answer?

Author response:

To ensure that the participants were not biased towards a specific answer, we used a few strategies:

First, in our questionnaire design, we used a 5-point Likert scale to provide balanced and comprehensive response options that allowed participants to express a full range of opinions.

Second, in our survey administration, we assured participants that their responses would remain anonymous and confidential to reduce the pressure to conform to socially desirable responses. We also provided clear instructions on how to answer the questions, emphasizing the importance of honest and accurate responses.

Third, we asked both positively and negatively worded questions, which helped us identify and control for the tendency of participants to agree with statements regardless of their content.

The first and third points are already mentioned in the manuscript. We have edited the “Survey instrument and data collection” section slightly to highlight the second point in lines 205-208 of the revised manuscript with track changes:

*“The participants were assured that their responses would remain anonymous and confidential to reduce the pressure to conform to socially desirable responses. Clear instructions were provided on how to answer the questions, emphasizing the importance of honest and accurate responses.”*

3. How the author makes sure that the participants were not disengaged while answering?

Author response:

To minimize the likelihood of participant disengagement during the questionnaire, we used clear and concise language that was easy to understand. We also grouped the questions that were related and avoided jumping between unrelated topics. Furthermore, we communicated at the start that the questionnaire would need 30-35 minutes to complete and their participation was important. This might have helped in managing their expectations upfront for a better engagement. Importantly, the focal points at each hospital kept track of the time taken to complete the survey. The focal points did not report any participants who might have sped through the questionnaire without careful consideration and completed the survey significantly faster than the average time of 30-35 minutes.

We have edited the text in the “Survey instrument and data collection” section to highlight this more in lines 191-192:

*“Clear and concise language was used for ease of understanding and questions that were related were grouped to avoid jumping between unrelated topics.”*

4. How the author makes sure about reliability of result?

Author response:

We used a valid and reliable survey tool and administered the survey in a standardized manner to all participants. In addition, we took a representative sample of healthcare workers in the province by a two-stage sampling technique to reduce sampling error and increase the likelihood that the results are generalizable and reliable. We also had an adequate sample size to detect reliable patterns and relationships in the data.

5. Author need to mention reliability and validity of tools used in the study

Author response:

The reliability and validity of the tool used in the study were assessed in a previous study in Thailand (Nakphook et al., 2024). The survey instrument was developed after a literature review using the adapted version of the vaccine hesitancy determinant matrix and finalized after completing qualitative focus group discussions. The validation process included expert review for content validity, pilot testing (n=30) for assessing feasibility, acceptability, and face validity, and a larger survey among a nationally representative sample of healthcare workers (n=2352) for evaluating construct validity and reliability. Construct validity was measured using an exploratory factor analysis (EFA) and internal consistency was assessed by Cronbach’s alpha. The final version of the survey instrument displayed good validity and acceptable reliability.

We have edited the text in the “Survey instrument and data collection” section to highlight this more in lines 195-200:

*“The study instrument was validated for use in the Thai context by another research project which used qualitative methods in the development of the questionnaire and had rigorous steps such as pilot testing (n=30) for assessing feasibility, acceptability, and face validity, and a larger survey among a nationally representative sample of healthcare workers (n=2352) for evaluating construct validity and reliability [40].”*

We have also added the online link of the validation paper in the reference no. 40 for easy access to the paper (lines 621-622).

6. Author need to mention response rate

Author response:

We have reported the participant’s response rate in the first section of the Results. We had an overall response rate of 96.6%. This has also been mentioned in Fig 1. The response rates varied at different hospitals, ranging from 80-100% which has been depicted in the supplementary table 2.

We hope that our changes will have addressed all the comments and meet with the approval of the reviewers.

Thank you for your kind consideration, and we look forward to hearing from you soon.

Best regards,

**Prabda Praphasiri,**

Corresponding Author
